# Robustness of CT radiomics features: consistency within and between single-energy CT and dual-energy CT

**DOI:** 10.1007/s00330-022-08628-3

**Published:** 2022-02-22

**Authors:** Yong Chen, Jingyu Zhong, Lan Wang, Xiaomeng Shi, Wei Lu, Jianying Li, Jianxing Feng, Yihan Xia, Rui Chang, Jing Fan, Liwei Chen, Ying Zhu, Fuhua Yan, Weiwu Yao, Huan Zhang

**Affiliations:** 1 Present Address: Department of Radiology, Ruijin Hospital, Shanghai Jiao Tong University School of Medicine, No. 197 Ruijin 2nd Road, Huangpu District, Shanghai, 200025 China; 2 Present Address: Department of Imaging, Tongren Hospital, Shanghai Jiao Tong University School of Medicine, No. 1111 Xianxia Road, Changning District, Shanghai, 200336 China; 3 Department of Materials, Imperial College London, South Kensington Campus, London, SW7 2AZ UK; 4 Computed Tomography Research Center, GE Healthcare, Shanghai, 201203 China; 5 Computed Tomography Research Center, GE Healthcare, Beijing, 100176 China; 6 Haohua Technology Co., Ltd., Shanghai, 201100 China

**Keywords:** Machine learning, Multidetector computed tomography, Reproducibility of results

## Abstract

**Objectives:**

To evaluate inter- and intra- scan mode and scanner repeatability and reproducibility of radiomics features within and between single-energy CT (SECT) and dual-energy CT (DECT).

**Methods:**

A standardized phantom with sixteen rods of clinical-relevant densities was scanned on seven DECT-capable scanners and three SECT-only scanners. The acquisition parameters were selected to present typical abdomen-pelvic examinations with the same voxel size. Images of SECT at 120 kVp and corresponding 120 kVp-like virtual monochromatic images (VMIs) in DECT which were generated according to scanners were analyzed. Regions of interest were drawn with rigid registrations to avoid variations due to segmentation. Radiomics features were extracted via Pyradiomics platform. Test-retest repeatability was evaluated by Bland-Altman analysis for repeated scans. Intra-scanner reproducibility for different scan modes was tested by intraclass correlation coefficient (ICC) and concordance correlation coefficient (CCC). Inter-scanner reproducibility among different scanners for same scan mode was assessed by coefficient of variation (CV) and quartile coefficient of dispersion (QCD).

**Results:**

The test-retest analysis presented that 92.91% and 87.02% of the 94 assessed features were repeatable for SECT 120kVp and DECT 120 kVp-like VMIs, respectively. The intra-scanner analysis for SECT 120kVp vs DECT 120 kVp-like VMIs demonstrated that 10.76% and 10.28% of features were with ICC > 0.90 and CCC > 0.90, respectively. The inter-scanner analysis showed that 17.09% and 27.73% of features for SECT 120kVp were with CV < 10% and QCD < 10%, and 15.16% and 32.78% for DECT 120 kVp-like VMIs, respectively.

**Conclusions:**

The majority of radiomics features were non-reproducible within and between SECT and DECT.

**Key Points:**

• *Although the test-retest analysis showed high repeatability for radiomics features, the overall reproducibility of radiomics features within and between SECT and DECT was low.*

• *Only about one-tenth of radiomics features extracted from SECT images and corresponding DECT images did match each other, even their average photon energy levels were considered alike, indicating that the scan mode potentially altered the radiomics features.*

• *Less than one-fifth of radiomics features were reproducible among multiple SECT and DECT scanners, regardless of their fixed acquisition and reconstruction parameters, suggesting the necessity of scanning protocol adjustment and post-scan harmonization process.*

**Supplementary Information:**

The online version contains supplementary material available at 10.1007/s00330-022-08628-3.

## Introduction

Radiomics refers to a workflow consisting conversion of digital medical images to mineable high-dimensional data, and whose subsequent analysis aims to support clinical decision-making [1–3]. The potential of radiomics in precision medicine has been pointed out [2], but the generalizability of the model and robustness of radiomics features were the main concern [4–7]. In contrast to other omics data, the robustness of radiomics features is influenced by multiple factors through the workflow, including data acquisition, image reconstruction, segmentation, image processing, and radiomics feature computation [5, 6]. Indeed, imaging devices and protocols have been demonstrated to significantly affect radiomic features in single-energy CT (SECT), MRI, and PET [8–10].

Dual-energy CT (DECT), with a second x-ray spectrum, allows the differentiation of multiple materials and generation of a set of virtual monochromatic images (VMIs) with an additional attenuation measurement, which makes possible several new and clinically relevant CT applications [11]. Radiomics has been applied to analyze the images from DECT, and showed convincible diagnostic and prognostic performance in oncology settings [12–14]. However, the factors associated with robustness of radiomics features in DECT have not been fully investigated. Only intensity discretization [15] and the energy levels of VMIs [16] were demonstrated as sources of uncertainty of radiomics features in DECT. It is necessary to systematically evaluate the inter- and intra-scanner robustness of radiomics features in both SECT and DECT modes to allow further multi-scanner investigations. For prospective studies with various DECT scanners, harmonizing upstream acquisition parameters can minimize the impact of imaging protocols [17]. Meanwhile, retrospective studies usually based on archived images from various SECT and DECT scans. It is important to determine whether those images are comparable enough as a basis for generating radiomics models for clinical decision-making.

Therefore, we aimed to evaluate inter- and intra-scan mode and scanner repeatability and reproducibility of radiomics features within and between SECT and DECT.

## Materials and methods

### Phantoms and CT scanners

**Fig. 1:**
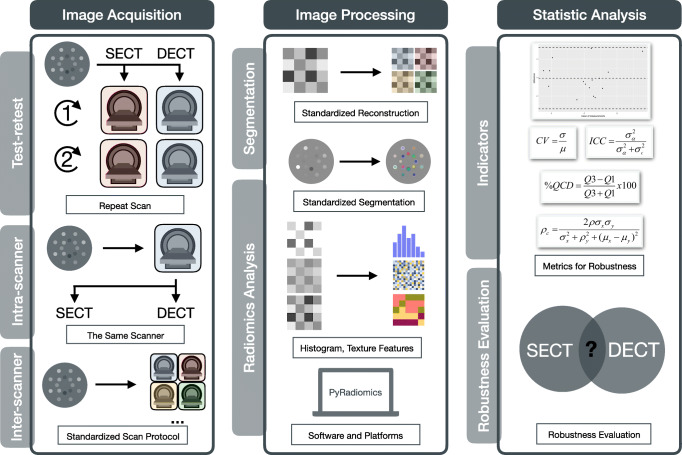
Study workflow. The study consisted three steps, namely image acquisition, image processing, and statistical analysis. A standardized phantom was scanned on seven DECT-capable scanners and three SECT-only scanners with the same voxel and typical abdomen-pelvic examination parameters. Eighteen first-order and 76 texture radiomics analysis were extracted by Pyradiomics platform from ROIs segmented with a rigid registration. Test-retest repeatability was evaluated by Bland-Altman analysis for repeated scans of the same scan mode, intra-scanner reproducibility for different scan modes was tested by ICC and CCC, and inter-scanner reproducibility among different scanners for same scan mode was tested by CV and QCD. The detailed description of the equations is available in the Supplementary Materials. *CCC*, concordance correlation coefficient; *CV*, coefficient of variation; *DECT*, dual-energy CT; *ICC*, intraclass correlation coefficient; *QCD*, quartile coefficient of dispersion; *ROI*, regions of interest; *SECT*, single-energy CT; *VMI* virtual monochromatic image

**Fig. 2:**
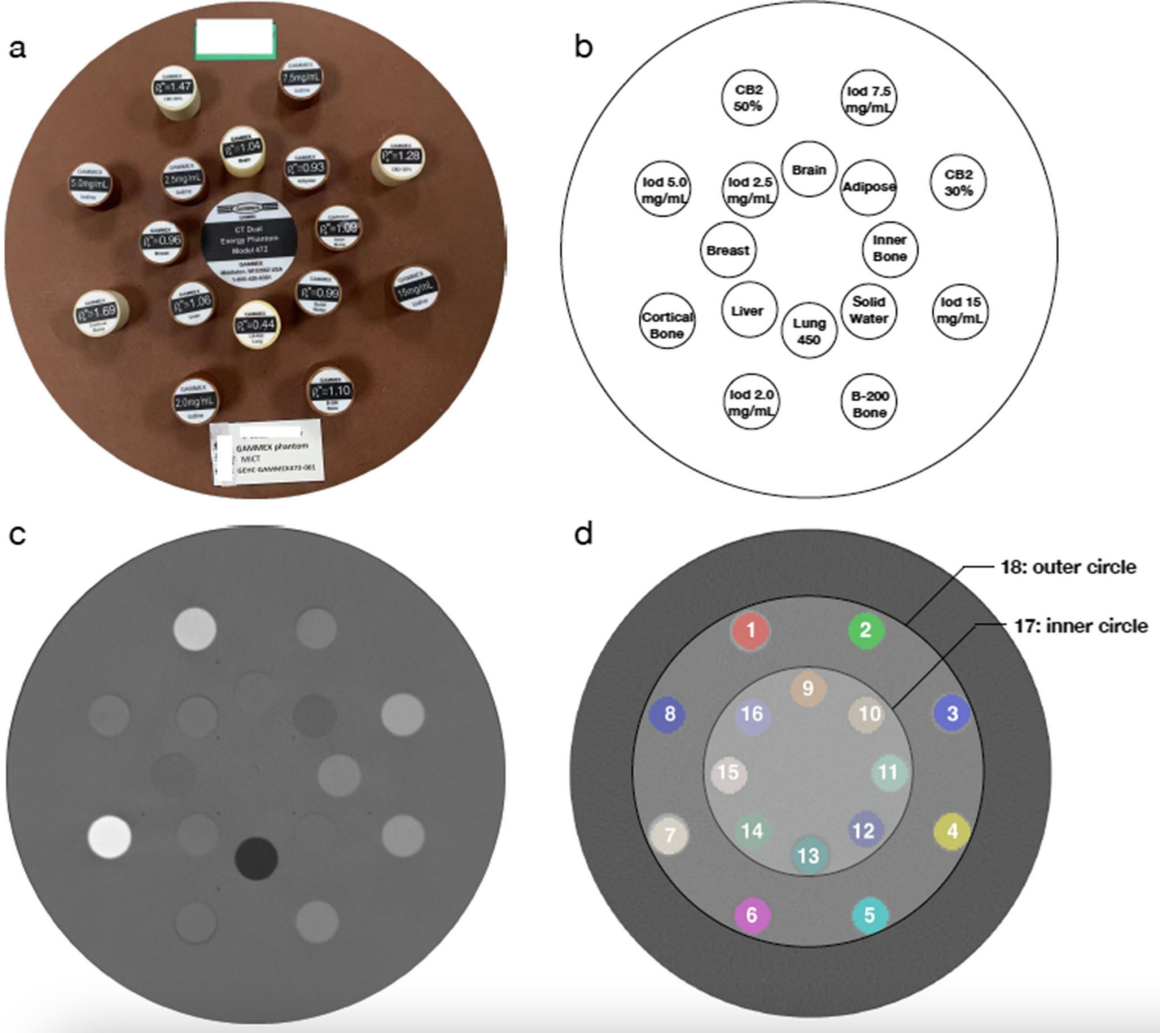
Phantom and segmentation. **a** The CT Dual Energy Phantom Model. **b** Sixteen rods of multiple clinical-relevant densities, including five rods with iodine (Iod) concentrations of 2.0 mg/mL, 2.5 mg/mL, 5.0 mg/mL, 7.5 mg/mL, and 15 mg/mL, and eleven rods with human body densities, namely lung (0.44 g/cm^3^), adipose (0.93 g/cm^3^), breast (0.96 g/cm^3^), solid water (0.99 g/cm^3^), brain (1.04 g/cm^3^), liver (1.06 g/cm^3^), inner bone (1.09 g/cm^3^), bone (1.10 g/cm^3^), cortical bone (CB) 2-30% (1.28 g/cm^3^), cortical bone (CB) 2-50% (1.47 g/cm^3^), and cortical bone (1.69 g/cm^3^). **c** Example CT scan. **d** Segmentation

The workflow of our study is shown in Fig. 1. Institutional review board approval was not required since only phantom was used. We used a CT Dual Energy Phantom Model (Gmamex 472, Gammex Inc.) consisting of a disk of 330 mm in diameter with water density and sixteen holes of 28 mm in diameter for holding interchangeable rods with various densities within the disk (Fig. 2a). We chose five iodine rods with concentrations from 2.0 to 15 mg/mL, and eleven tissue rods with densities of 0.44 to 1.69 g/cm^3^, to give us a wide range of Hounsfield unit (HU) values (Fig. 2b). The position of the rods was chosen to minimize beam-hardening artifacts and was kept the same throughout the scans in the study.

**Fig. 3:**
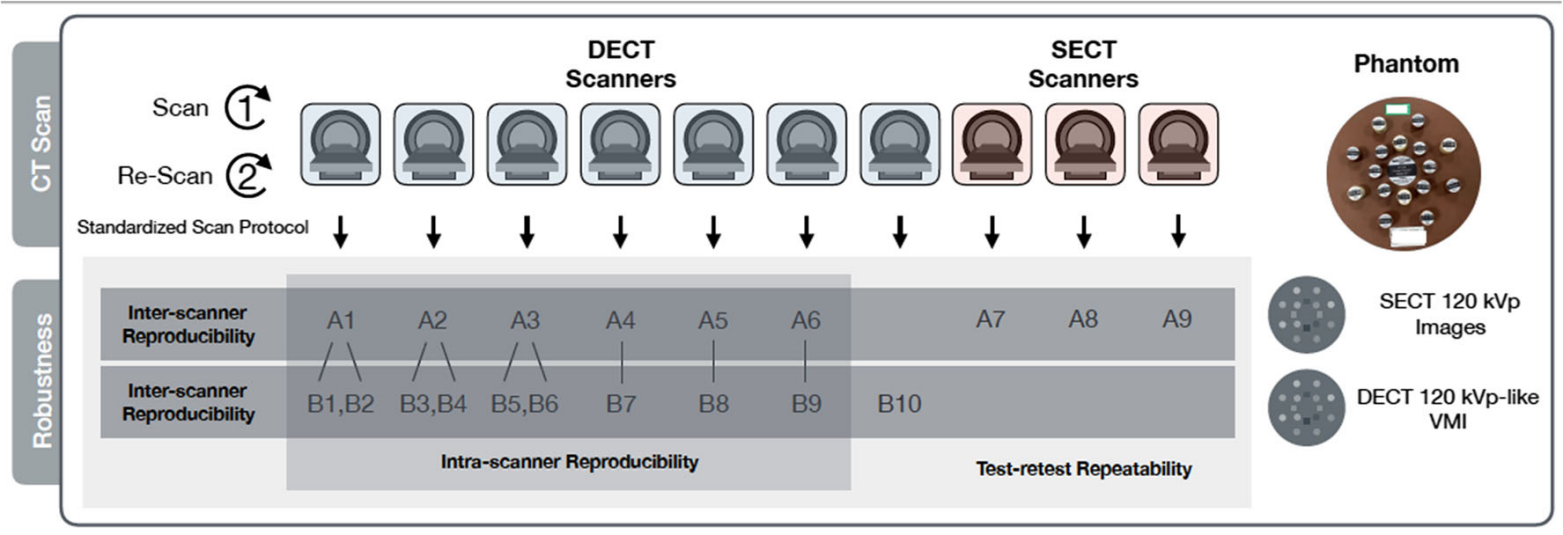
CT scan and robustness analysis. The phantom was imaged with seven DECT-capable scanners and three SECT-only scanners. Group A refers to SECT 120 kVp images, group B refers to DECT 120 kVp-like VMIs. Test-retest repeatability was evaluated by Bland-Altman analysis for repeated scans of the same scan mode. The first three DECT scanners generated two sets of DECT images each using two different tube voltage combinations, and the other three DECT scanners generated one set of DECT images each. Therefore, there were nine pairs of intra-scanner reproducibility tested by ICC and CCC. The inter-scanner reproducibility among different scanners for same scan mode was assessed by CV and QCD. *CCC*, concordance correlation coefficient; *CV*, coefficient of variation; *DECT* dual-energy CT; *ICC*, intraclass correlation coefficient; *QCD*, quartile coefficient of dispersion; *SECT* single-energy CT; *VMI* virtual monochromatic image

The phantom was scanned on seven DECT-capable scanners and three SECT-only scanners in two centers (Fig. 3), with comparable scan parameters. Each scan was repeated after repositioning, several minutes apart, to allow robustness analysis. The scanners and acquisition parameters are described in Table 1. To keep the voxel size stable, the field of view (50.0 × 50.0 cm), reconstruction matrix (512 × 512), and slice thickness (5 cm) remained unchanged for all acquisitions. The tube voltage, volume CT dose index, iteration reconstruction method, and reconstruction kernel were selected to present typical abdomen-pelvic examinations.

**Table 1:** CT acquisition parameters

No.	Vendor	Scanner	Type	Tube voltage (kVp)	Milliamperage (mA or mAs)	Rotation time (sec)	volume CT dose index (mGy)	Iteration method	Reconstruction kernel	Images
A1	SIEMENS	SOMATOM Dirve	SECT	120	313	1.0	19.99	ADMIRE 2	I40s	SECT 120 kVp
A2	SIEMENS	SOMATOM Definition Flash	SECT	120	296	1.0	19.97	SAFIRE 2	I40s	SECT 120 kVp
A3	SIEMENS	SOMATOM Force	SECT	120	299	1.0	19.99	ADMIRE 2	Br40	SECT 120 kVp
A4	GE	Discovery CT750 HD	SECT	120	345^*^	0.8	20.05	ASiR-V 40	Standard	SECT 120 kVp
A5	GE	Revolution Apex	SECT	120	215^*^	0.7	19.98	ASiR-V 40	Standard	SECT 120 kVp
A6	GE	Revolution CT	SECT	120	190^*^	0.8	20.00	ASiR-V 40	Standard	SECT 120 kVp
A7	UI	uCT 760	SECT	120	238	1.0	20.02	KARL 3D 4	Abdomen	SECT 120 kVp
A8	GE	LightSpeed	SECT	120	235*	1.0	20.03	FBP	Standard	SECT 120 kVp
A9	PHILIPS	Brillance	SECT	120	309	0.75	20.00	FBP	Standard (B)	SECT 120 kVp
B1	SIEMENS	SOMATOM Dirve	DSCT	80/140	580/224	0.5	20.00	ADMIRE 2	Q40f	DECT 120 kVp-like
B2	SIEMENS	SOMATOM Dirve	DSCT	100/140	279/216	0.5	20.04	ADMRE 2	Q40f	DECT 120 kVp-like
B3	SIEMENS	SOMATOM Definition Flash	DSCT	80/140	531/205	1.0	20.01	SAFIRE 2	Q40s	DECT 120 kVp-like
B4	SIEMENS	SOMATOM Definition Flash	DSCT	100/140	258/199	1.0	19.96	SAFIRE 2	Q40s	DECT 120 kVp-like
B5	SIEMENS	SOMATOM Force	DSCT	70/150	848/212	0.5	20.00	ADMIRE 2	Qr40	DECT 120 kVp-like
B6	SIEMENS	SOMATOM Force	DSCT	100/150	294/147	0.5	20.02	ADMIRE 2	Qr40	DECT 120 kVp-like
B7	GE	Discovery CT750 HD	KVSCT	80/140	640^*^	0.6	21.84	ASiR-V 40	Standard	DECT 120 kVp-like
B8	GE	Revolution Apex	KVSCT	80/140	370^*^	1.0	19.75	ASiR-V 40	Standard	DECT 120 kVp-like
B9	GE	Revolution CT	KVSCT	80/140	275^*^	0.8	20.00	ASiR-V 40	Standard	DECT 120 kVp-like
B10	PHILIPS	IQon spectral CT	DLCT	120	221	0.75	20.00	iDOSE 3	Standard (B)	DECT 120 kVp-like

*mA not mAs for GE medical systems

*DECT* dual-energy CT, *DLCT* dual-layer detector CT, *DSCT* dual-source CT, *KVSCT* rapid kV-switching CT, *SECT* single-energy CT, *VMI* virtual monoenergetic image

Images of single-tube voltage at 120 kV (SECT 120kVp) were acquired on six DECT scanners and three SECT scanners (A1 to A9). Images of the corresponding DECT images with similar photon energy level to the SECT 120-kVp images (DECT 120 kVp-like VMIs) were generated on seven DECT scanners. Three of the seven DECT scanners provide two dual-energy scan modes using different tube voltage combinations, and six sets of DECT images were obtained with these three DECT scanners (B1 to B6). The other four DECT scanners generated one set of DECT images each (B7 to B10). Therefore, there were ten sets of corresponding DECT images in total (B1 to B10). For dual-source DECT, data acquired with two energy spectra (at two different tube voltages) were used to create a weighted average or a blend of images to simulate a kilovolt peak level of 120 kVp. For rapid kV-switching DECT and dual-layer detector DECT, the VMIs at appropriate kiloelectron voltage levels that mimic the average photon energy levels of the 120-kVp X-ray spectrum were selected. These kinds of DECT images were selected because they were usually used for radiomics analysis in daily research practice.

### Segmentation and feature extraction

We drew the regions of interest (ROIs) by using an open-source software ITK-SNAP version 3.6.0 (http://www.itksnap.org/pmwiki/pmwiki.php). To minimize variations in image segmentation, we copied the ROIs from one examination to another. Eighteen ROIs were selected in our study (Fig. 2d). Sixteen ROIs (ROI 1 to 16) were circles of 25 mm (26 pixels) in diameter set at the center of each rod, to cover each rod as much as possible, and avoid to touch its edge. The other two ROIs (ROI 17 and 18) were circles of 146 mm (150 pixels) and 244 mm (250 pixels) in diameter, centering at the disk and covering 8 and 16 rods, respectively, to present the mixed densities in human body.

Python version 3.7.6 (https://www.python.org) with Pyradiomics package version 3.0 (https://pyradiomics.readthedocs.io/en/latest/) was used to extract the radiomics features based on the original images, including 18 first-order features and 76 texture features. Twenty-six shape-based features were excluded since the shape of ROIs was consistent. Therefore, there were 94 radiomics features extracted from each ROI.

### Test-retest repeatability analysis for radiomics features

For test-retest analysis, all repeated scans were involved (Fig. 3). Radiomics features were extracted on 18 ROIs of the middle three layers of images from two repeating scans with the same acquisition parameters on the same scanner. The ROIs were copied from the first scan to the second to preclude ROI variations. The repeatability was assessed by Bland-Altman analysis [18, 19]. The percentage of repeatable features were calculated with cutoff values of 0.85, 0.90, and 0.95.

### Intra-scanner reproducibility analysis for radiomics features

Schematics of the intra-scanner reproducibility test which evaluates the consistency between two different scan modes (SECT vs DECT) of each scanner is shown in Fig. 3. Since the first three DECT-capable scanners generated two sets of DECT images each, there were six pairs of intra-scanner images compared (A1 vs B1, A1 vs B2, A2 vs B3, A2 vs B4, A3 vs B5, and A3 vs B6). The other three DECT-capable scanners generated one set of DECT images each, and three pairs of comparison were performed (A4 vs B7, A5 vs B8, and A6 vs B9). Therefore, there were nine pairs of comparisons in total. The concordance correlation coefficient (CCC) [20, 21] and the intraclass correlation coefficient (ICC) using single rater, absolute agreement, two-way random effects model [22] was employed as comparative measures with cutoff values of 0.85, 0.90, and 0.95.

### Inter-scanner reproducibility for radiomics features

The inter-scanner reproducibility analysis was performed among SECT 120-kVp images (A1 to A9), and among DECT 120 kVp-like VMIs (B1 to B10) (Fig. 3). The coefficient of variation (CV) [23] and quartile coefficient of dispersion (QCD) [24] with cutoff values of 5%, 10%, and 15% were used as measures to evaluate the reproducibility. The correlation between reproducibility and material density was not analyzed since the scatter plots did not show potential correlations (Supplementary Figures S1 and S2).

### Intra- and inter-scanner reproducibility for CT number values

To investigate whether there was any consistency between radiomics features and CT numbers, we also performed intra- and inter-scanner reproducibility analysis of CT numbers for 18 ROIs. Difference of CT number values and standard deviation (SD) of each ROI between SECT and DECT mode within the same scanner was also compared.

### Statistical analysis

The statistical analysis was performed by using R language version 3.6.3 (https://www.r-project.org/) with DescTools version 0.99.41 and BlandAltmanLeh version 0.3.1 packages. Comparison of continuous variables between two groups was performed by independent *t* test. We also generated heatmaps of ICC, CCC, CV, and QCD to assess the robustness of radiomics features across the scanners and scan modes. The two-sided *p* < 0.05 was considered statistically significant. The Bonferroni method was used to correct for multiple comparisons (*p* = 0.05/8 = 0.006). The detailed formula for analysis is available in the Supplementary Materials S2.

## Results

### Test-retest repeatability analysis for radiomics features

The average percentage ± SD of repeatable radiomics features was 92.91 ± 1.89% in SECT 120-kVp images and 87.02 ± 5.79% in DECT 120 kVp-like VMIs, when the cutoff was 0.90. Overall, 89.81 ± 4.47% of radiomics features were considered repeatable. The test-retest analysis showed high repeatability with various cutoffs (Supplementary Table S1).

### Intra-scanner reproducibility analysis for radiomics features

For intra-scanner reproducibility of SECT 120-kVp images vs DECT 120 kVp-like VMIs, the average percentage ± SD of intra-scanner reproducible radiomics features of ROI 1 to 16 was 10.76 ± 2.05% for ICC > 0.90, and 10.28 ± 2.05% for CCC > 0.90 (Table 2). Likewise, the corresponding average percentage ± SD of reproducible radiomics features of ROI 17 and 18 was 51.30 ± 9.19% and 40.54 ± 10.06%, respectively, for ICC > 0.90 and CCC > 0.90. Further, the intra-scanner reproducibility of ROI 17 and 18 was demonstrated to be higher than that of ROI 1 to 16 for ICC > 0.90 and CCC > 0.90 (both *p* < 0.001). The analysis resulted in low intra-scanner reproducibility, according to ICC and CCC with various cutoffs, and the average ICC and CCC (Table 2, Supplementary S2).

**Table 2:** Intra-scanner reproducibility analysis for radiomics features

ROI	ICC > 0.85	ICC > 0.90	ICC > 0.95	ICC mean	CCC > 0.85	CCC > 0.90	CCC > 0.95	CCC mean
ROI 1 to 16	13.95%	10.76%	8.16%	0.4731	13.59%	10.28%	7.92%	0.4632
ROI 17 and 18	57.57%	51.30%	39.48%	0.7081	48.11%	40.54%	29.20%	0.6704
*p* value for ROI 1 to 16 vs ROI 17 and 18	< 0.001	< 0.001	< 0.001	< 0.001	< 0.001	< 0.001	< 0.001	< 0.001

Percentage indicates the percentage of features met the cutoffs for repeatable measures (ICC > 0.90 and CCC > 0.90)

*CCC* concordance correlation coefficient, *ICC* intraclass correlation coefficient, *ROI* region of interest

### Inter-scanner reproducibility for radiomics features

For inter-scanner reproducibility of ROI 1 to 16, the average percentage ± SD of radiomics features meeting the criteria of CV < 10% and QCD < 10% was 17.09 ± 2.60% and 27.73 ± 4.07% for SECT 120-kVp images, and 15.16 ± 3.26% and 31.78 ± 5.62% for DECT 120 kVp-like VMIs, respectively (Table 3). For inter-scanner reproducibility of ROI 17 and 18, the results reached 47.87 ± 0.00% and 61.70 ± 0.00%, and 38.30 ± 6.38% and 55.32 ± 1.06%, respectively, with CV < 10% and QCD < 10%. Further, the inter-scanner reproducibility of ROI 17 and 18 was higher than that of ROI 1 to 16 by CV < 10% and QCD < 10%; in both SECT and DECT images (all *p* < 0.001), there was no significant difference in the inter-scanner reproducibility between SECT and DECT images (*p* = 0.39 for ICC, *p* = 0.44 for CCC). The inter-scanner reproducibility analysis demonstrated significant variation of radiomics features, by CV and QCD with various cutoffs, and the average CV and QCD (Table 3, Supplementary Table S3).

**Table 3:** Inter-scanner reproducibility analysis for radiomics features

ROI	Material	SECT 120 kVp Images				DECT 120 kVp-like VMIs			
		CV < 10%	CV mean	QCD < 10%	QCD mean	CV < 10%	CV mean	QCD < 10%	QCD mean
Iodine concentration									
6	Iodine (2.0 mg/mL)	15.96%	0.4209	26.60%	0.1916	13.83%	0.3716	31.91%	0.2306
16	Iodine (2.5 mg/mL)	13.83%	0.5509	21.28%	0.2891	12.77%	0.3779	20.21%	0.2342
8	Iodine (5.0 mg/mL)	9.57%	0.3444	24.47%	0.2470	13.83%	0.2859	37.23%	0.1693
2	Iodine (7.5 mg/mL)	15.96%	0.4223	25.53%	0.2669	15.96%	0.2752	41.49%	0.1657
4	Iodine (15 mg/mL)	20.21%	0.4816	27.66%	0.2817	12.77%	0.3094	41.49%	0.1793
Human body density									
13	Lung (0.44 g/cm^3^)	23.40%	0.3198	37.23%	0.2014	24.47%	0.5834	34.04%	0.3804
10	Adipose (0.93 g/cm^3^)	17.02%	0.3655	28.72%	0.2495	21.28%	0.3997	24.47%	0.2979
15	Breast (0.96 g/cm^3^)	20.21%	0.3527	27.66%	0.2317	19.15%	0.5704	35.11%	0.2233
12	Solid Water (0.99 g/cm^3^)	17.02%	0.4507	22.34%	0.2882	17.02%	0.7775	28.72%	0.8327
9	Brain (1.04 g/cm^3^)	13.83%	0.4054	21.28%	0.2410	13.83%	0.3865	23.40%	0.2433
14	Liver (1.06 g/cm^3^)	13.83%	1.5921	20.21%	0.2207	19.15%	0.5890	39.36%	0.2323
11	Inner Bone (1.09 g/cm^3^)	18.09%	0.3407	28.72%	0.2190	12.77%	0.3116	31.91%	0.1890
5	Bone (1.10 g/cm^3^)	18.09%	2.9275	31.91%	0.2653	13.83%	0.3962	32.98%	0.6981
3	CB2-30% (1.28 g/cm^3^)	19.15%	0.4434	32.98%	0.2406	12.77%	0.3354	37.23%	0.1916
1	CB2-50% (1.47 g/cm^3^)	15.96%	1.6117	27.66%	0.4099	11.70%	0.4300	24.47%	0.2472
7	Cortical Bone (1.69 g/cm^3^)	21.28%	0.4281	39.36%	0.4044	7.45%	0.6213	24.47%	0.3540
ROI 17 and 18									
17	8 rods	47.87%	0.2314	61.70%	0.3222	44.68%	0.2034	56.38%	0.1201
18	16 rods	47.87%	0.1795	61.70%	0.1354	31.91%	0.2189	54.26%	0.1121
Overall		17.09%	0.7161	27.73%	0.2655	15.16%	0.4388	31.78%	0.3043
*p* value for ROI 1 to 16 vs ROI 17 and 18		< 0.001	0.34	< 0.001	0.49	< 0.001	0.046	< 0.001	0.20

Percentage indicates the percentage of features met the cutoffs for repeatable measures (CV < 10% and QCD < 10%)

*CV* coefficient of variation, *DECT* dual-energy CT, *QCD* quartile coefficient of dispersion, *SECT* single-energy CT, *ROI* region of interest, *VMI* virtual monoenergetic image

### Robust radiomics features

Heatmaps of ICC, CCC, CV, and QCD were drawn to assess what features are more robust across the scanners and scan modes (Supplementary Figures S3 and S4). The visual assessment suggested that the first-order features were more likely to be reproducible than texture features.

### Intra- and inter-scanner reproducibility for CT number values

The CT number values of ROIs and their variations are available in Supplementary Tables S4 to S8. Overall, the CT number values varied between SECT and DECT even in the same scanner and varied among scanners from different vendors. The differences of CT number values between SECT and DECT were from 9.86 to 111.90 HU. The difference between SECT and DECT in dual-source DECT scanners (B1 to B6, CT number difference 9.86 to 111.90 HU) seemed to be dependent on different tube voltage combinations, and the rapid kV-switching DECT scanners (B7 to B9, CT number difference 17.65 to 27.85 HU) were relatively stable. The small variations were relatively small in ROI 1 to 16 (mean SD 8.54 to 17.45 HU in SECT, 9.28 to 23.91 HU in DECT) and relatively great in ROI 17 and 18 (mean SD 50.17 and 85.97 HU in SECT, 51.91 and 94.64 HU in DECT).

## Discussion

Our study, for the first time, evaluated the test-retest repeatability, intra-scanner reproducibility between different scan modes, and inter-scanner reproducibility of radiomics features in SECT and DECT, by using a phantom with rods of clinical-relevant multiple densities. Our results demonstrated that the test-retest repeatability was acceptable, but the inter- and intra-scan mode and scanner reproducibility were relatively low. The intra-scanner reproducibility analysis demonstrated that the radiomics features extracted from SECT 120-kVp images and DECT 120 kVp-like VMIs did not match each other, even though they were acquired on the same scanner with fixed parameters, and images had similar average photon energy. The inter-scanner reproducibility suggested wide variation of radiomics features extracted from both SECT 120 kVp images and DECT 120 kVp-like VMIs among different scanners. However, correlations between inter-scanner reproducibility and material density were not detected. Additionally, we found that the first-order features were more likely to be reproducible than texture features (Supplementary Figures S3 and S4).

The intra-scanner reproducibility analysis indicated that SECT 120-kVp images and DECT 120 kVp-like VMIs were far from alike from the radiomics features point of view. The images generated from various DECT scanners differed from those from conventional SECT because of differences in their acquisition techniques, material decomposition methods, image reconstruction algorithms, and postprocessing methods [25]. Although SECT-like images were generated in DECT to mimic the SECT images, the intra-scanner reproducibility of radiomics features was low between SECT images and corresponding SECT-like images in DECT. Regarding the fixed acquisition and processing parameters, the intra-scanner variation might reflect the influence of different technique approaches between SECT and DECT. Our analysis further indicated that CT number values varied significantly among scanners and scan modes, and the intra-scanner CT number value difference between SECT and DECT might be a source of variation (Supplementary Figures S5 and S6). Further investigations on the SECT and DECT energy dependency of radiomics features are needed. Considering the large variation of CT number values among scanners and scan modes, the small variations of raw input might not be the main source of radiomics variation. Investigations on the influence of the small variations of CT number values might be possible in the future, when stable CT number values were available among scanners and scan modes. Since the majority of SECT and DECT radiomics features were not reproducible in the same scanner, it is necessary to interpret them with caution, especially in retrospective studies where consistency of acquisition parameters was not available. Our results also provided insights for the adjustment of imaging protocols in prospective study design, that involvement of images from both SECT and DECT scanners might need extra correction procedure.

The inter-scanner reproducibility analysis mainly reflects the variations among vendors and scanners. Many steps in radiomics analysis have specific drawbacks that would need to be resolved. For instance, the robustness of radiomics features could vary due to data acquisition, image reconstruction, segmentation, and feature extraction [8, 26–30]. The change of voxel size could lead to the increase of radiomics features variability [26]. Therefore, in our study, we made the field of view, reconstruction matrix, and slice thickness the same for different scanners during acquisition, to keep the voxel the same. Since radiation dose influences on the reproducibility [27], the tube voltage, milliamperage, and rotation time were carefully adjusted to maintain the volume CT dose index similar among scans. A rigid registration was employed to translate ROIs, avoiding the variation due to delineations [28]. All the radiomics features were extracted via Pyradiomics, an Image Biomarker Standardisation Initiative compliant platform [29, 31], with harmonized calculation settings, to minimize the influence of feature extraction platform. Unfortunately, several parameters could hardly be uniformed among different scanners. We selected reconstruction kernels and iteration method of a typical abdominal-pelvic examination, to allow comparable results among scanners [27, 30], but most of them were vendor-dependent, and impossible to harmonize. Further, CT number values vary across scanners due to the different X-ray spectra of different scanners [32], which might lead to differences in radiomics features. Additional slight differences of the images caused by different calibrations methods could be translated in radiomics variability [8]. In addition, the introduction of DECT scanners made it more difficult to reach a high reproducibility among scanners. The best energy level for VMI reconstruction to match the SECT image differs among vendors. Therefore, corresponding DECT images have different imaging appearances, texture features, and quantitative capabilities [25]. Further, different technical approaches to realize DECT, namely dual-source DECT, dual-layer detector DECT, and rapid kV-switching DECT, might potentially be unique sources of variability in our study [11, 25], resulting in low inter-scanner reproducibility of radiomics features.

Acquisition parameters have greatly affected the reproducibility of radiomics features in SECT, MRI, and PET [8–10]. Our study further showed that the approaches that generate similar DECT images corresponding to SECT images might yield images with different texture characteristics, because the imaging techniques used differ among vendors and scanners. The factors associated with the robustness of radiomics features in DECT have been rarely investigated. Chatterjee et al [15] performed voxel intensity discretization through four binning algorithms, and showed the impact of HU value range on radiomics feature stability using DECT data. Baliyan et al [16] demonstrated that the energy levels of VMIs have different impacts on the texture analysis. These sources of uncertainty are recommended to take into account when evaluating the robustness of radiomics features in DECT images in order to increase the likelihood of replicability. Overall, we consider that the main source of radiomics variation might be a combination of SECT and DECT difference, and varying CT number values among scanners.

Berenguer et al [8] found that the reproducibility of radiomics features depended on the kind of material, in which the densest wood showed the highest reproducibility. Differences of reproducibility among sixteen rods were observed in our study, but the correlations between reproducibility and material density were not evident. Notably, two ROIs covering rods with various density showed higher intra- and inter-scanner reproducibility than those of sixteen uniform rods. As a phantom study, its non-validated nature causes concern. So far, the Credence Cartridge Radiomics phantom is the one most used for radiomics investigation [33, 34], which provides cartridges with different textures and CT number values. However, all the scans of this phantom were performed on SECT scanners. In contrast, the phantom used in our study is dedicated for DECT quality assurance, and has been scanned on both SCET and DECT scanners. The Credence Cartridge Radiomics phantom is composed of acrylonitrile butadiene styrene, acrylic beads, and polyvinyl chloride, which might not be the best to present human body, while our model could present the physiological situation of multiple tissues using clinical-relevant densities. Further, we drew ROI 1 to 16 to present the homogeneous human tissues, and ROI 17 and 18 to present the human body with mixed densities. Radiomics features might be more robust in image with more obvious structural feature, which also matched our finding that first-order features were more likely to be reproducible than texture features. We hypothesize that small variations of input data might have greater influence on the homogenous ROIs. Further investigations are under consideration to validate this hypothesis.

There were several limitations in our study. First, our study did not test a wide range of acquisition parameters to be comprehensive and generalizable [8], but rather chose the imaging protocol to present a typical abdomen-pelvic examination to be more translatable to the clinical practice. Second, we only compared the SECT 120-kVp images and DECT 120 kVp-like VMIs to present daily research practice. We selected vendor-recommended 120 kVp-like DECT images, and showed that their intra-scanner reproducibility was low, but it is worth investigating the true equivalent energy levels to generate VMIs in DECT, which could be object-dependent with high intra- and inter-scanner reproducibility with SECT images. Third, radiomics features can be expanded by extracting from images with wavelet or Laplacian of Gaussian transformations, but we only evaluated those extracted from the original images. We did not include the images with filtering or transformation, because of the image processing effects on the reproducibility of radiomics features [35], which was not the aim of our work. Fourth, various feature extraction platforms have been developed for radiomics investigations; of those, we employed the Pyradiomics platform for radiomics feature extraction which is considered a reliable tool for radiomics feature extraction in phantom and clinical studies [31]. We kept the settings harmonized during the feature extraction, but it is unknown how the feature extraction platforms influence the robustness in DECT radiomics. Fifth, we used a DECT phantom with homogenous rods for scanning. Comparing to the radiomics phantom [33, 34], ours might lack texture. However, the phantom allows more specific results in human benefiting by its similarity to human density. Lastly, as a phantom study, our results could not be directly translated into clinical practice. Due to the highly homogenous nature of the phantom, our results could not fully reflect the characteristics of real disease. Moreover, our results must not be compared with those of clinical predictive studies. Nonetheless, our study emphasized the intra-scanner difference between SECT and DECT technique, to which attention should be paid in future investigations. Meanwhile, the reproducibility could be impaired if insufficient image processing were conducted to combat inter-scanner variability [17].

In summary, our study indicated that the radiomics features extracted from SECT images and corresponding DECT images did not match each other, even if their average photon energy levels were considered alike. The majority of radiomics features were not reproducible among scanners, even if multiple acquisition parameters were fixed. The first-order features were more likely to be reproducible than texture features, and might provide an opportunity for improving robustness of radiomics models. Radiomics results from multiple CT scanners and with different scan techniques must be interpreted with caution because of potential risks of non-reproducible data.

## Supplementary Information

ESM 1 (DOCX 2.83 mb)

## Abbreviations

CCC: Concordance correlation coefficient
CV: Coefficient of variation
DECT: Dual-energy CT
HU: Hounsfield units
ICC: Intraclass correlation coefficient
QCD: Quartile coefficient of dispersion
ROI: Region of interest
SD: Standard deviation
SECT: Single-energy CT
VMI: Virtual monochromatic image

## Declarations

### Guarantor

The scientific guarantor of this publication is Prof. Huan Zhang from the Department of Radiology, Ruijin Hospital, Shanghai Jiao Tong University School of Medicine.

### Statistics and biometry

No complex statistical methods were necessary for this paper.

### Methodology

• Experimental

• Multicenter study
