# Supplementary material for: Robustness of CT radiomics features: consistency within and between single-energy CT and dual-energy CT
Source: Eur Radiol. 2022 Feb 22;32(8):5480–90. doi: 10.1007/s00330-022-08628-3 (PMC9279234; doi:10.1007/s00330-022-08628-3)
Supplement: Supplementary file 1 — (DOCX 2.83 mb) [file 330_2022_8628_MOESM1_ESM.docx]

**Supplementary Materials**

**Title:** Robustness of CT Radiomics Features: Consistency within and between Single-energy CT and Dual-energy CT

**List of Supplementary Materials**

Supplementary Method

Supplementary Tables

Supplementary Table S1 Test-retest Repeatability Analysis

Supplementary Table S2 Intra-scanner Reproducibility Analysis

Supplementary Table S3 Inter-scanner Reproducibility Analysis

Supplementary Table S4 CT number values of 18 ROIs in SECT 120 kVp A1-A9 image sets

Supplementary Table S5 CT number values of 18 ROIs in DECT 120 kVp-like VMI B1-B10 images sets

Supplementary Table S6 Difference of CT number values between SECT and DECT mode within the same scanner

Supplementary Table S7 SD values of 18 ROIs in SECT 120 kVp A1-A9 image sets

Supplementary Table S8 SD values of 18 ROIs in DECT 120 kVp-like VMI B1-B10 images sets

Supplementary Figures

Supplementary Figure S1 Iodine Concentrate and Inter-scanner Reproducibility

Supplementary Figure S2 Material Density and Inter-scanner Reproducibility

Supplementary Figure S3 Heatmap of ICC and CCC

Supplementary Figure S4 Heatmap of CV and QCD

Supplementary Figure S5 Intra-scanner SECT/DECT CT number value difference and ICC/CCC mean value

Supplementary Figure S6 Intra-scanner SECT/DECT CT number value difference and percentage of ICC/CCC > 0.90

**Supplementary Method**

The statistical analysis was performed by using R language version 3.6.3 (<https://www.r-project.org/>) with DescTools version 0.99.41 and BlandAltmanLeh version 0.3.1 packages. The formulae used in our study followed the Berenguer et al [1].

**CV formula:**


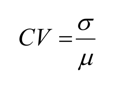


CV is defined as the ratio of the standard deviation (σ) to the mean (μ) [2].

**QCD formula:**

**
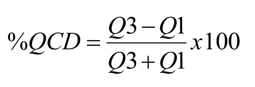
**

where Q1 and Q3 are the first and third quartiles, respectively [3].

**CCC formula:**


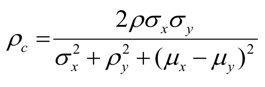


where μ_x_ and μ_y_ are the means for the 2 variables and σ^2^_x_ and σ^2^_y_ are the corresponding variances. Ρ is the correlation coefficient between the two variables [4,5].

**ICC formula:**


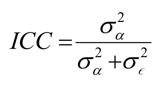


where σ^2^_α_ is the pooled variance within the subjects, and σ2ε is the trait between subjects [6].

**Reference:**

[1] Berenguer R, Pastor-Juan MDR, Canales-Vázquez J, et al. Radiomics of CT features may be nonreproducible and redundant: influence of CT acquisition parameters. Radiology 2018;288(2):407-415.

[2] Reed GF, Lynn F, Meade BD. Use of coefficient of variation in assessing variability of quantitative assays. Clin Diagn Lab Immunol 2002;9(6):1235–1239.

[3] Bonett DG. Confidence interval for a coefficient of quartile variation. Comput Stat Data Anal 2006;50(11):2953–2957.

[4] Lin LI. A concordance correlation coefficient to evaluate reproducibility. Biometrics 1989;45(1):255–268.

[5] Lin LI. A note on the concordance correlation coefficient. Biometrics 2000;56(1):324–325.

[6] Koo TK, Li MY. A Guideline of Selecting and Reporting Intraclass Correlation Coefficients for Reliability Research. J Chiropr Med 2016;15(2):155–163.

**Supplementary Table S1 Test-retest Repeatability Analysis**

| No | B-A > 0.85 | B-A > 0.90 | B-A > 0.95 |
| --- | --- | --- | --- |
| A1 | 100.00% | 92.55% | 42.55% |
| A2 | 100.00% | 92.55% | 35.11% |
| A3 | 100.00% | 89.36% | 38.30% |
| A4 | 100.00% | 92.55% | 42.55% |
| A5 | 100.00% | 97.87% | 22.34% |
| A6 | 100.00% | 90.43% | 22.34% |
| A7 | 100.00% | 93.62% | 21.28% |
| A8 | 100.00% | 91.49% | 21.28% |
| A9 | 100.00% | 95.74% | 24.47% |
| A1-A9 | 100.00% | 92.91% | 30.02% |
| B1 | 100.00% | 76.60% | 27.66% |
| B2 | 100.00% | 93.62% | 30.85% |
| B3 | 100.00% | 90.43% | 32.98% |
| B4 | 100.00% | 97.87% | 29.79% |
| B5 | 100.00% | 91.49% | 29.79% |
| B6 | 100.00% | 81.91% | 17.02% |
| B7 | 100.00% | 88.30% | 32.98% |
| B8 | 100.00% | 89.36% | 11.70% |
| B9 | 100.00% | 85.11% | 30.85% |
| B10 | 100.00% | 75.53% | 15.96% |
| B1-B10 | 100.00% | 87.02% | 25.96% |
| Overall | 100.00% | 89.81% | 27.88% |

Note: B-A indicates percentage of features met the criteria of Bland-Altman analysis.

**Supplementary Table S2 Intra-scanner Reproducibility Analysis**

|  | ICC > 0.85 | ICC > 0.90 | ICC > 0.95 | ICC mean | CCC > 0.85 | CCC > 0.90 | CCC > 0.95 | CCC mean |
| --- | --- | --- | --- | --- | --- | --- | --- | --- |
| ROI 1 to 16 |  |  |  |  |  |  |  |  |
| A1 vs B1 | 15.96% | 12.77% | 7.45% | 0.506271 | 15.96% | 11.70% | 7.45% | 0.497039 |
| A1 vs B2 | 9.57% | 9.57% | 9.57% | 0.436434 | 9.57% | 9.57% | 9.57% | 0.426189 |
| A2 vs B3 | 21.28% | 14.89% | 10.64% | 0.544074 | 21.28% | 14.89% | 8.51% | 0.533700 |
| A2 vs B4 | 11.70% | 10.64% | 9.57% | 0.599166 | 11.70% | 10.64% | 9.57% | 0.586427 |
| A3 vs B5 | 7.45% | 4.26% | 0.00% | 0.297309 | 7.45% | 3.19% | 0.00% | 0.287783 |
| A3 vs B6 | 12.77% | 9.57% | 7.45% | 0.520597 | 11.70% | 9.57% | 7.45% | 0.510761 |
| A4 vs B7 | 19.15% | 10.64% | 9.57% | 0.403355 | 18.09% | 9.57% | 9.57% | 0.395582 |
| A5 vs B8 | 10.64% | 10.64% | 9.57% | 0.352485 | 10.64% | 10.64% | 9.57% | 0.344722 |
| A6 vs B9 | 17.02% | 13.83% | 9.57% | 0.598281 | 15.96% | 12.77% | 9.57% | 0.586761 |
| Overall | 13.95% | 10.76% | 8.16% | 0.473108 | 13.59% | 10.28% | 7.92% | 0.463218 |
| ROI 17 and 18 |  |  |  |  |  |  |  |  |
| A1 vs B1 | 54.26% | 43.62% | 26.60% | 0.744586 | 42.55% | 30.85% | 20.21% | 0.681038 |
| A1 vs B2 | 57.45% | 46.81% | 39.36% | 0.730261 | 45.74% | 39.36% | 36.17% | 0.685135 |
| A2 vs B3 | 57.45% | 53.19% | 30.85% | 0.700055 | 48.94% | 34.04% | 14.89% | 0.642660 |
| A2 vs B4 | 70.21% | 62.77% | 54.26% | 0.757206 | 58.51% | 54.26% | 44.68% | 0.747459 |
| A3 vs B5 | 35.11% | 22.34% | 19.15% | 0.596843 | 20.21% | 19.15% | 10.64% | 0.539645 |
| A3 vs B6 | 53.19% | 52.13% | 41.49% | 0.629381 | 51.06% | 42.55% | 27.66% | 0.656653 |
| A4 vs B7 | 56.38% | 51.06% | 34.04% | 0.669267 | 42.55% | 34.04% | 25.53% | 0.618120 |
| A5 vs B8 | 68.09% | 64.89% | 55.32% | 0.788823 | 62.77% | 56.38% | 51.06% | 0.750898 |
| A6 vs B9 | 65.96% | 64.89% | 54.26% | 0.756762 | 60.64% | 54.26% | 31.91% | 0.711615 |
| Overall | 57.57% | 51.30% | 39.48% | 0.708132 | 48.11% | 40.54% | 29.20% | 0.670358 |
| P-value for ROI 1-16 vs ROI 17 and 18 | <0.0001 | <0.0001 | <0.0001 | <0.0001 | <0.0001 | <0.0001 | 0.0003 | 0.0001 |

Abbreviations: CCC = concordance correlation coefficient, DECT = dual energy CT, ICC = intraclass correlation coefficient, SECT = single energy CT, ROI = reigion of interest, VMI = virtual monoenergetic image.

**Supplementary Table S3 Inter-scanner Reproducibility Analysis**

| Type | ROI | Material | CV<15% | CV<10% | CV<5% | CV mean | QCD<15% | QCD<10% | QCD<5% | QCD mean |
| --- | --- | --- | --- | --- | --- | --- | --- | --- | --- | --- |
| SECT 120 kVp  (A1-A9) | 6 | Iodine (2.0 mg/mL) | 14.89% | 15.96% | 6.38% | 0.420944 | 53.19% | 26.60% | 12.77% | 0.191568 |
|  | 16 | Iodine (2.5 mg/mL) | 12.77% | 13.83% | 6.38% | 0.550920 | 32.98% | 21.28% | 11.70% | 0.289097 |
|  | 8 | Iodine (5.0 mg/mL) | 18.09% | 9.57% | 5.32% | 0.344429 | 31.91% | 24.47% | 10.64% | 0.246962 |
|  | 2 | Iodine (7.5 mg/mL) | 20.21% | 15.96% | 5.32% | 0.422341 | 38.30% | 25.53% | 13.83% | 0.266936 |
|  | 4 | Iodine (15 mg/mL) | 21.28% | 20.21% | 4.26% | 0.481586 | 39.36% | 27.66% | 13.83% | 0.281720 |
|  | 13 | Lung (0.44 g/cm^3^) | 24.47% | 23.40% | 15.96% | 0.319758 | 47.87% | 37.23% | 21.28% | 0.201417 |
|  | 10 | Adipose (0.93 g/cm^3^) | 19.15% | 17.02% | 11.70% | 0.365506 | 37.23% | 28.72% | 17.02% | 0.249452 |
|  | 15 | Breast (0.96 g/cm^3^) | 20.21% | 20.21% | 13.83% | 0.352681 | 45.74% | 27.66% | 17.02% | 0.231709 |
|  | 12 | Solid Water (0.99 g/cm^3^) | 12.77% | 17.02% | 9.57% | 0.450663 | 36.17% | 22.34% | 14.89% | 0.288196 |
|  | 9 | Brain (1.04 g/cm^3^) | 15.96% | 13.83% | 7.45% | 0.405383 | 32.98% | 21.28% | 9.57% | 0.240983 |
|  | 14 | Liver (1.06 g/cm^3^) | 19.15% | 13.83% | 4.26% | 1.592085 | 41.49% | 20.21% | 10.64% | 0.220717 |
|  | 11 | Inner Bone (1.09 g/cm^3^) | 24.47% | 18.09% | 5.32% | 0.340742 | 42.55% | 28.72% | 14.89% | 0.218984 |
|  | 5 | Bone (1.10 g/cm^3^) | 28.72% | 18.09% | 5.32% | 2.927546 | 51.06% | 31.91% | 22.34% | 0.265289 |
|  | 3 | CB2-30% (1.28 g/cm^3^) | 25.53% | 19.15% | 8.51% | 0.443357 | 43.62% | 32.98% | 21.28% | 0.240595 |
|  | 1 | CB2-50% (1.47 g/cm^3^) | 23.40% | 15.96% | 10.64% | 1.611672 | 41.49% | 27.66% | 18.09% | 0.409867 |
|  | 7 | Cortical Bone (1.69 g/cm^3^) | 27.66% | 21.28% | 5.32% | 0.428135 | 53.19% | 39.36% | 19.15% | 0.404415 |
|  | 17 | 8 rods | 59.57% | 47.87% | 29.79% | 0.2313613 | 70.21% | 61.70% | 40.43% | 0.322249 |
|  | 18 | 16 rods | 61.70% | 47.87% | 26.60% | 0.1794709 | 72.34% | 61.70% | 42.55% | 0.135400 |
|  | - | Overall | 20.55% | 17.09% | 7.85% | 0.7161091 | 41.82% | 27.73% | 15.56% | 0.265494 |
|  | P-value for ROI 1-16 vs ROI 17 and 18 | | <0.0001 | <0.0001 | < 0.0001 | 0.3419 | <0.0001 | <0.0001 | <0.0001 | 0.4873 |
| DECT 120 kVp-like  (B1-B10) | 6 | Iodine (2.0 mg/mL) | 18.09% | 13.83% | 6.38% | 0.371626 | 47.87% | 31.91% | 13.83% | 0.230647 |
|  | 16 | Iodine (2.5 mg/mL) | 13.83% | 12.77% | 5.32% | 0.377905 | 44.68% | 20.21% | 7.45% | 0.234152 |
|  | 8 | Iodine (5.0 mg/mL) | 19.15% | 13.83% | 5.32% | 0.285947 | 54.26% | 37.23% | 14.89% | 0.169275 |
|  | 2 | Iodine (7.5 mg/mL) | 25.53% | 15.96% | 6.38% | 0.275216 | 63.83% | 41.49% | 19.15% | 0.165718 |
|  | 4 | Iodine (15 mg/mL) | 21.28% | 12.77% | 6.38% | 0.309424 | 61.70% | 41.49% | 11.70% | 0.179296 |
|  | 13 | Lung (0.44 g/cm^3^) | 26.60% | 24.47% | 13.83% | 0.583444 | 54.26% | 34.04% | 15.96% | 0.380350 |
|  | 10 | Adipose (0.93 g/cm^3^) | 20.21% | 21.28% | 11.70% | 0.399711 | 41.49% | 24.47% | 18.09% | 0.297886 |
|  | 15 | Breast (0.96 g/cm^3^) | 21.28% | 19.15% | 11.70% | 0.570398 | 54.26% | 35.11% | 20.21% | 0.223292 |
|  | 12 | Solid Water (0.99 g/cm^3^) | 18.09% | 17.02% | 11.70% | 0.777512 | 50.00% | 28.72% | 12.77% | 0.832677 |
|  | 9 | Brain (1.04 g/cm^3^) | 12.77% | 13.83% | 6.38% | 0.386530 | 42.55% | 23.40% | 11.70% | 0.243329 |
|  | 14 | Liver (1.06 g/cm^3^) | 26.60% | 19.15% | 5.32% | 0.588992 | 59.57% | 39.36% | 17.02% | 0.232316 |
|  | 11 | Inner Bone (1.09 g/cm^3^) | 23.40% | 12.77% | 6.38% | 0.311555 | 51.06% | 31.91% | 13.83% | 0.189031 |
|  | 5 | Bone (1.10 g/cm^3^) | 25.53% | 13.83% | 6.38% | 0.396195 | 45.74% | 32.98% | 14.89% | 0.698130 |
|  | 3 | CB2-30% (1.28 g/cm^3^) | 22.34% | 12.77% | 5.32% | 0.335411 | 51.06% | 37.23% | 19.15% | 0.191584 |
|  | 1 | CB2-50% (1.47 g/cm^3^) | 21.28% | 11.70% | 5.32% | 0.430026 | 44.68% | 24.47% | 14.89% | 0.247238 |
|  | 7 | Cortical Bone (1.69 g/cm^3^) | 14.89% | 7.45% | 5.32% | 0.621254 | 34.04% | 24.47% | 7.45% | 0.353968 |
|  | 17 | 8 rods | 52.13% | 44.68% | 21.28% | 0.2034404 | 75.53% | 56.38% | 37.23% | 0.120119 |
|  | 18 | 16 rods | 42.55% | 31.91% | 13.83% | 0.2188962 | 75.53% | 54.26% | 30.85% | 0.112123 |
|  | - | Overall | 20.68% | 15.16% | 7.45% | 0.4388215 | 50.07% | 31.78% | 14.56% | 0.304305 |
|  | P-value for ROI 1-16 vs ROI 17 and 18 | | <0.0001 | <0.0001 | 0.0005 | 0.0466 | 0.0004 | 0.0002 | <0.0001 | 0.1950 |
| P-value for SECT 120 kVp vs DECT 120 kVp-like | | | 0.7342 | 0.3930 | 0.4557 | 0.1518 | 0.0465 | 0.4395 | 0.5411 | 0.6469 |

Abbreviations: CV = coefficient of variation, DECT = dual energy CT, QCD = quartile coefficient of dispersion, SECT = single energy CT, ROI = region of interest, VMI = virtual monoenergetic image.

**Table S4 CT number values of 18 ROIs in SECT 120 kVp A1-A9 image sets**

| ROI | A1 | A2 | A3 | A4 | A5 | A6 | A7 | A8 | A9 | Mean | SD |
| --- | --- | --- | --- | --- | --- | --- | --- | --- | --- | --- | --- |
| 1 | 754.939 | 776.060 | 823.553 | 851.098 | 872.839 | 855.708 | 814.462 | 864.235 | 826.122 | 826.557 | 40.075 |
| 2 | 141.422 | 150.149 | 163.063 | 187.370 | 166.848 | 161.913 | 160.270 | 182.434 | 169.453 | 164.769 | 14.315 |
| 3 | 405.181 | 425.198 | 455.546 | 482.057 | 471.790 | 465.137 | 449.488 | 469.054 | 468.420 | 454.652 | 24.734 |
| 4 | 265.835 | 283.029 | 315.331 | 351.410 | 338.739 | 332.884 | 314.414 | 340.051 | 325.614 | 318.590 | 28.002 |
| 5 | 180.272 | 191.896 | 220.728 | 223.193 | 226.865 | 225.389 | 216.803 | 214.329 | 222.363 | 213.538 | 16.306 |
| 6 | 27.833 | 32.734 | 46.698 | 48.601 | 39.187 | 43.490 | 42.781 | 36.430 | 52.521 | 41.142 | 7.890 |
| 7 | 1104.253 | 1141.804 | 1231.399 | 1247.570 | 1337.844 | 1312.592 | 1216.963 | 1227.410 | 1248.197 | 1229.781 | 73.195 |
| 8 | 98.476 | 103.445 | 111.234 | 138.082 | 106.963 | 108.851 | 108.060 | 138.407 | 125.923 | 115.493 | 14.871 |
| 9 | 21.310 | 17.711 | 25.121 | 36.675 | 19.227 | 20.492 | 17.874 | 25.918 | 43.408 | 25.304 | 8.989 |
| 10 | -81.031 | -84.081 | -82.291 | -65.267 | -94.620 | -91.783 | -88.089 | -73.354 | -65.139 | -80.628 | 10.721 |
| 11 | 184.128 | 185.635 | 210.417 | 227.037 | 213.408 | 211.232 | 198.030 | 220.946 | 215.386 | 207.358 | 14.983 |
| 12 | -9.019 | -11.506 | 0.786 | 5.673 | -4.892 | -2.203 | -5.478 | -10.331 | 1.289 | -3.965 | 5.834 |
| 13 | -506.722 | -518.358 | -534.170 | -504.835 | -537.491 | -535.177 | -526.429 | -510.622 | -526.994 | -522.311 | 12.630 |
| 14 | 66.512 | 62.782 | 76.989 | 84.706 | 65.584 | 71.267 | 68.082 | 67.679 | 77.932 | 71.281 | 7.139 |
| 15 | -42.392 | -48.212 | -43.074 | -23.823 | -54.918 | -51.478 | -48.112 | -44.313 | -32.698 | -43.224 | 9.636 |
| 16 | 53.246 | 48.715 | 62.260 | 74.417 | 50.041 | 53.476 | 53.691 | 58.689 | 74.587 | 58.791 | 9.803 |
| 17 | -17.419 | -19.744 | -9.943 | 0.611 | -17.247 | -14.444 | -15.589 | -15.275 | 1.292 | -11.973 | 7.797 |
| 18 | 30.157 | 32.613 | 42.192 | 56.187 | 39.818 | 41.775 | 39.278 | 41.439 | 52.752 | 41.801 | 8.348 |

Note: present as HU values.

Abbreviations: CV = coefficient of variation, HU = Hounsfield unit, SD = standard deviation, ROI = region of interest.

**Table S5 CT number values of 18 ROIs in DECT 120 kVp-like VMI B1-B10 images sets**

| ROI | B1 | B2 | B3 | B4 | B5 | B6 | B7 | B8 | B9 | B10 | Mean | SD |
| --- | --- | --- | --- | --- | --- | --- | --- | --- | --- | --- | --- | --- |
| 1 | 868.492 | 776.176 | 885.364 | 790.101 | 1212.073 | 954.941 | 842.635 | 920.337 | 942.529 | 877.970 | 902.797 | 116.844 |
| 2 | 176.939 | 140.446 | 183.862 | 147.014 | 273.645 | 173.277 | 174.980 | 193.326 | 198.422 | 185.068 | 183.239 | 34.746 |
| 3 | 473.959 | 428.607 | 486.334 | 439.437 | 637.407 | 514.131 | 466.462 | 498.188 | 507.065 | 498.953 | 492.409 | 55.149 |
| 4 | 345.670 | 274.477 | 357.979 | 286.498 | 549.495 | 358.581 | 344.642 | 395.563 | 401.216 | 378.699 | 366.915 | 72.208 |
| 5 | 231.348 | 201.929 | 237.447 | 209.530 | 325.685 | 240.161 | 205.662 | 244.100 | 253.492 | 238.155 | 237.243 | 33.789 |
| 6 | 48.071 | 37.838 | 50.141 | 40.758 | 74.098 | 46.999 | 27.287 | 45.789 | 52.294 | 47.249 | 46.277 | 11.630 |
| 7 | 1308.326 | 1164.812 | 1328.209 | 1185.816 | 1909.521 | 1471.767 | 1300.062 | 1402.253 | 1443.897 | 1291.116 | 1373.559 | 201.229 |
| 8 | 120.459 | 94.682 | 123.997 | 98.576 | 178.985 | 112.615 | 121.543 | 124.509 | 132.247 | 122.682 | 121.728 | 22.191 |
| 9 | 19.820 | 26.747 | 15.612 | 24.176 | 8.462 | 26.797 | 17.912 | 7.519 | 21.736 | 27.895 | 18.772 | 7.577 |
| 10 | -87.457 | -81.555 | -89.501 | -84.378 | -105.178 | -87.476 | -82.928 | -99.069 | -94.226 | -78.140 | -89.569 | 8.147 |
| 11 | 221.500 | 194.107 | 221.588 | 194.724 | 307.304 | 227.128 | 213.168 | 230.409 | 240.396 | 243.076 | 227.464 | 30.992 |
| 12 | 0.195 | -0.652 | -1.824 | -2.703 | 4.509 | 2.975 | -16.356 | -6.389 | 1.971 | 13.427 | -1.377 | 7.861 |
| 13 | -531.873 | -534.331 | -535.377 | -538.552 | -536.795 | -530.640 | -516.693 | -533.164 | -538.705 | -539.618 | -533.760 | 6.342 |
| 14 | 75.969 | 75.142 | 73.050 | 72.274 | 75.550 | 77.316 | 65.953 | 67.101 | 78.038 | 78.691 | 73.099 | 4.950 |
| 15 | -45.713 | -42.962 | -50.194 | -46.597 | -58.055 | -46.899 | -42.473 | -57.216 | -50.995 | -38.380 | -48.607 | 6.359 |
| 16 | 64.837 | 52.390 | 61.669 | 49.163 | 85.617 | 60.072 | 53.173 | 58.081 | 69.299 | 79.034 | 62.183 | 11.795 |
| 17 | -9.835 | -11.435 | -12.274 | -13.966 | -4.921 | -8.556 | -18.216 | -17.492 | -10.362 | -2.839 | -11.726 | 5.251 |
| 18 | 45.045 | 37.344 | 46.052 | 38.514 | 71.837 | 49.989 | 38.205 | 43.447 | 50.138 | 50.192 | 46.249 | 9.905 |

Note: present as HU values.

Abbreviations: CV = coefficient of variation, HU = Hounsfield unit, SD = standard deviation, ROI = region of interest.

**Table S6 Difference of CT number values between SECT and DECT mode within the same scanner**

| ROI | A1-B1 | A1-B2 | A2-B3 | A2-B4 | A3-B5 | A3-B6 | A4-B7 | A5-B8 | A6-B9 | Mean | SD |
| --- | --- | --- | --- | --- | --- | --- | --- | --- | --- | --- | --- |
| 1 | 113.553 | 21.237 | 109.304 | 14.041 | 388.52 | 131.388 | 8.463 | 47.498 | 86.821 | 102.314 | 116.915 |
| 2 | 35.517 | 0.976 | 33.713 | 3.135 | 110.582 | 10.214 | 12.39 | 26.478 | 36.509 | 29.946 | 33.276 |
| 3 | 68.778 | 23.426 | 61.136 | 14.239 | 181.861 | 58.585 | 15.595 | 26.398 | 41.928 | 54.661 | 51.881 |
| 4 | 79.835 | 8.642 | 74.95 | 3.469 | 234.164 | 43.25 | 6.768 | 56.824 | 68.332 | 64.026 | 70.561 |
| 5 | 51.076 | 21.657 | 45.551 | 17.634 | 104.957 | 19.433 | 17.531 | 17.235 | 28.103 | 35.909 | 28.824 |
| 6 | 20.238 | 10.005 | 17.407 | 8.024 | 27.4 | 0.301 | 21.314 | 6.602 | 8.804 | 13.344 | 8.666 |
| 7 | 204.073 | 60.559 | 186.405 | 44.012 | 678.122 | 240.368 | 52.492 | 64.409 | 131.305 | 184.638 | 199.043 |
| 8 | 21.983 | 3.794 | 20.552 | 4.869 | 67.751 | 1.381 | 16.539 | 17.546 | 23.396 | 19.757 | 19.848 |
| 9 | 1.49 | 5.437 | 2.099 | 6.465 | 16.659 | 1.676 | 18.763 | 11.708 | 1.244 | 7.282 | 6.816 |
| 10 | 6.426 | 0.524 | 5.42 | 0.297 | 22.887 | 5.185 | 17.661 | 4.449 | 2.443 | 7.255 | 7.792 |
| 11 | 37.372 | 9.979 | 35.953 | 9.089 | 96.887 | 16.711 | 13.869 | 17.001 | 29.164 | 29.558 | 27.401 |
| 12 | 9.214 | 8.367 | 9.682 | 8.803 | 3.723 | 2.189 | 22.029 | 1.497 | 4.174 | 7.742 | 6.228 |
| 13 | 25.151 | 27.609 | 17.019 | 20.194 | 2.625 | 3.53 | 11.858 | 4.327 | 3.528 | 12.871 | 9.952 |
| 14 | 9.457 | 8.63 | 10.268 | 9.492 | 1.439 | 0.327 | 18.753 | 1.517 | 6.771 | 7.406 | 5.786 |
| 15 | 3.321 | 0.57 | 1.982 | 1.615 | 14.981 | 3.825 | 18.65 | 2.298 | 0.483 | 5.303 | 6.682 |
| 16 | 11.591 | 0.856 | 12.954 | 0.448 | 23.357 | 2.188 | 21.244 | 8.04 | 15.823 | 10.722 | 8.552 |
| 17 | 7.584 | 5.984 | 7.47 | 5.778 | 5.022 | 1.387 | 18.827 | 0.245 | 4.082 | 6.264 | 5.335 |
| 18 | 14.888 | 7.187 | 13.439 | 5.901 | 29.645 | 7.797 | 17.982 | 3.629 | 8.363 | 12.092 | 8.053 |
| Mean | 40.086 | 12.524 | 36.961 | 9.861 | 111.699 | 30.541 | 18.374 | 17.650 | 27.849 | n/a | n/a |
| SD | 50.964 | 14.608 | 47.041 | 10.228 | 173.983 | 61.570 | 9.514 | 19.741 | 35.368 | n/a | n/a |

Note: present as HU values.

Abbreviations: CV = coefficient of variation, HU = Hounsfield unit, SD = standard deviation, ROI = region of interest, n/a = not applicable.

**Table S7 SD values of 18 ROIs in SECT 120 kVp A1-A9 image sets**

| ROI | A1 | A2 | A3 | A4 | A5 | A6 | A7 | A8 | A9 | HU mean | SD mean |
| --- | --- | --- | --- | --- | --- | --- | --- | --- | --- | --- | --- |
| 1 | 14.818 | 17.489 | 12.968 | 10.032 | 10.006 | 9.719 | 6.885 | 17.578 | 13.570 | 826.557 | 12.563 |
| 2 | 10.890 | 12.190 | 8.927 | 11.347 | 7.781 | 8.674 | 7.647 | 11.501 | 12.565 | 164.769 | 10.169 |
| 3 | 11.391 | 13.608 | 8.760 | 7.502 | 7.168 | 7.226 | 6.364 | 11.379 | 12.289 | 454.652 | 9.521 |
| 4 | 10.311 | 12.271 | 8.437 | 8.746 | 7.442 | 7.920 | 6.811 | 11.189 | 12.556 | 318.590 | 9.520 |
| 5 | 9.445 | 11.146 | 8.372 | 7.845 | 6.533 | 6.944 | 6.061 | 10.592 | 11.460 | 213.538 | 8.711 |
| 6 | 9.032 | 10.293 | 8.556 | 7.305 | 7.035 | 6.528 | 7.225 | 9.348 | 11.489 | 41.142 | 8.535 |
| 7 | 17.328 | 21.619 | 19.514 | 17.101 | 17.223 | 15.438 | 8.767 | 20.605 | 19.436 | 1229.781 | 17.448 |
| 8 | 11.166 | 12.666 | 10.079 | 8.273 | 6.818 | 7.348 | 7.724 | 12.251 | 14.028 | 115.493 | 10.039 |
| 9 | 10.879 | 12.159 | 9.034 | 8.565 | 6.833 | 7.657 | 7.764 | 14.442 | 15.422 | 25.304 | 10.306 |
| 10 | 10.229 | 12.093 | 7.701 | 7.943 | 6.154 | 6.717 | 6.691 | 11.227 | 12.574 | -80.628 | 9.037 |
| 11 | 11.071 | 12.567 | 9.270 | 9.265 | 7.657 | 7.130 | 7.036 | 13.110 | 14.151 | 207.358 | 10.140 |
| 12 | 9.752 | 11.686 | 8.355 | 8.557 | 6.356 | 7.389 | 6.690 | 14.196 | 13.346 | -3.965 | 9.592 |
| 13 | 10.781 | 12.308 | 10.394 | 8.635 | 7.339 | 7.627 | 7.938 | 11.288 | 13.637 | -522.311 | 9.994 |
| 14 | 10.481 | 11.641 | 9.444 | 9.473 | 7.851 | 6.405 | 6.710 | 12.272 | 14.173 | 71.281 | 9.828 |
| 15 | 10.275 | 11.675 | 9.197 | 8.302 | 7.030 | 6.698 | 6.591 | 11.358 | 13.014 | -43.224 | 9.349 |
| 16 | 11.282 | 13.207 | 9.305 | 8.395 | 6.386 | 6.563 | 6.841 | 14.109 | 13.477 | 58.791 | 9.952 |
| 17 | 48.290 | 49.608 | 50.997 | 49.490 | 50.360 | 50.413 | 48.863 | 50.983 | 52.524 | -11.973 | 50.170 |
| 18 | 78.772 | 81.035 | 86.162 | 87.887 | 90.868 | 89.057 | 84.607 | 89.977 | 85.319 | 41.801 | 85.965 |

Note: present as HU values.

Abbreviations: CV = coefficient of variation, HU = Hounsfield unit, SD = standard deviation, ROI = region of interest.

**Table S8 SD values of 18 ROIs in DECT 120 kVp-like VMI B1-B10 images sets**

| ROI | B1 | B2 | B3 | B4 | B5 | B6 | B7 | B8 | B9 | B10 | HU mean | SD mean |
| --- | --- | --- | --- | --- | --- | --- | --- | --- | --- | --- | --- | --- |
| 1 | 11.150 | 10.071 | 16.181 | 13.209 | 28.488 | 15.968 | 12.263 | 9.634 | 9.721 | 13.321 | 902.797 | 14.001 |
| 2 | 10.081 | 9.550 | 13.126 | 11.324 | 12.864 | 10.078 | 8.430 | 9.087 | 6.986 | 10.660 | 183.239 | 10.219 |
| 3 | 9.946 | 9.297 | 13.822 | 11.807 | 13.581 | 10.649 | 7.601 | 7.854 | 6.920 | 10.193 | 492.409 | 10.167 |
| 4 | 10.194 | 9.192 | 13.842 | 11.943 | 14.658 | 9.722 | 9.085 | 8.397 | 7.358 | 9.929 | 366.915 | 10.432 |
| 5 | 9.893 | 9.239 | 13.582 | 11.836 | 11.311 | 9.256 | 7.019 | 7.169 | 7.033 | 9.093 | 237.243 | 9.543 |
| 6 | 9.489 | 8.627 | 13.434 | 11.405 | 10.689 | 9.496 | 8.779 | 7.746 | 8.287 | 8.596 | 46.277 | 9.655 |
| 7 | 15.465 | 13.262 | 22.573 | 16.602 | 67.950 | 28.884 | 21.529 | 18.231 | 14.483 | 20.155 | 1373.559 | 23.913 |
| 8 | 10.436 | 9.629 | 13.425 | 11.761 | 11.863 | 9.101 | 9.102 | 9.767 | 8.719 | 10.805 | 121.728 | 10.461 |
| 9 | 10.110 | 9.626 | 13.243 | 12.011 | 10.932 | 8.308 | 7.689 | 7.653 | 8.431 | 12.045 | 18.772 | 10.005 |
| 10 | 10.056 | 9.398 | 13.071 | 11.553 | 9.480 | 8.136 | 6.759 | 6.724 | 6.527 | 11.058 | -89.569 | 9.276 |
| 11 | 10.979 | 10.391 | 14.722 | 12.782 | 12.650 | 10.268 | 8.947 | 8.227 | 7.901 | 10.987 | 227.464 | 10.785 |
| 12 | 10.081 | 9.901 | 13.940 | 12.291 | 10.048 | 8.335 | 7.470 | 7.852 | 7.103 | 10.317 | -1.377 | 9.734 |
| 13 | 10.811 | 10.162 | 13.168 | 12.310 | 10.536 | 9.412 | 7.852 | 8.049 | 7.603 | 9.346 | -533.760 | 9.925 |
| 14 | 10.176 | 9.929 | 14.438 | 12.500 | 10.825 | 8.364 | 8.867 | 7.748 | 7.420 | 11.320 | 73.099 | 10.159 |
| 15 | 10.180 | 9.694 | 13.790 | 12.129 | 10.440 | 8.264 | 8.201 | 6.935 | 6.747 | 9.721 | -48.607 | 9.610 |
| 16 | 10.583 | 10.498 | 13.999 | 12.505 | 11.084 | 8.889 | 8.407 | 8.446 | 7.234 | 10.826 | 62.183 | 10.247 |
| 17 | 51.517 | 50.605 | 53.389 | 51.760 | 55.632 | 51.064 | 48.981 | 50.477 | 52.101 | 53.546 | -11.726 | 51.907 |
| 18 | 90.788 | 80.163 | 92.579 | 81.873 | 125.483 | 96.151 | 90.270 | 97.683 | 99.254 | 92.162 | 46.249 | 94.641 |

Note: present as HU values.

Abbreviations: CV = coefficient of variation, HU = Hounsfield unit, SD = standard deviation, ROI = region of interest.

**Supplementary Figure S1 Iodine Concentrate and Inter-scanner Reproducibility**

SECT = single-energy CT, DECT = dual energy CT, CV = coefficient of variation, QCD = quartile coefficient of dispersion. Dot line indicates the result of liner regression.

**(A) SECT 120 kVp**

**(B) DECT 120 kVp-like**

**Supplementary Figure S2 Material Density and Inter-scanner Reproducibility**

SECT = single-energy CT, DECT = dual energy CT, CV = coefficient of variation, QCD = quartile coefficient of dispersion. Dot line indicates the result of liner regression.

**(A) SECT 120 kVp**

**(B) DECT 120 kVp-like**

**Supplementary Figure S3 Heatmap of ICC and CCC**

Blue indicates higher consistency, and red indicates higher variability. CCC = concordance correlation coefficient, ICC = intraclass correlation coefficient, GLCM = gray-level co-occurrence matrix, GLDM = gray level dependence matrix, GLRLM = gray-level run-length matrix, GLSZM = gray-level size zone matrix, NGTDM = neighborhood gray-tone difference matrix, ROI = region of interest.

**Supplementary Figure S4 Heatmap CV and QCD**

Blue indicates higher consistency, and white and red indicates higher variability. CV = coefficient of variation, GLCM = gray-level co-occurrence matrix, GLDM = gray level dependence matrix, GLRLM = gray-level run-length matrix, GLSZM = gray-level size zone matrix, NGTDM = neighborhood gray-tone difference matrix, QCD = quartile coefficient of dispersion, ROI = region of interest.

**Figure S5 Intra-scanner SECT/DECT CT number value difference and ICC/CCC mean value**

SECT = single-energy CT, DECT = dual energy CT, CCC = concordance correlation coefficient, ICC = intraclass correlation coefficient, HU = Hounsfield unit. Dot line indicates the result of liner regression.

**Figure S6 Intra-scanner SECT/DECT CT number value difference and percentage of ICC/CCC > 0.90**

SECT = single-energy CT, DECT = dual energy CT, CCC = concordance correlation coefficient, ICC = intraclass correlation coefficient HU = Hounsfield unit. Dot line indicates the result of liner regression.
